# In-Orbit Attitude Determination of the UVSQ-SAT CubeSat Using TRIAD and MEKF Methods

**DOI:** 10.3390/s21217361

**Published:** 2021-11-05

**Authors:** Adrien Finance, Christophe Dufour, Thomas Boutéraon, Alain Sarkissian, Antoine Mangin, Philippe Keckhut, Mustapha Meftah

**Affiliations:** 1 Université de Versailles Saint-Quentin-en-Yvelines, Université Paris-Saclay, Sorbonne Université (SU), CNRS, LATMOS, 11 Boulevard d’Alembert, 78280 Guyancourt, France; Adrien.Finance@latmos.ipsl.fr (A.F.); Christophe.Dufour@latmos.ipsl.fr (C.D.); Thomas.Bouteraon@latmos.ipsl.fr (T.B.); Alain.Sarkissian@latmos.ipsl.fr (A.S.); Philippe.Keckhut@latmos.ipsl.fr (P.K.); 2 ACRI-ST CERGA, 10 Avenue Nicolas Copernic, 06130 Grasse, France; Antoine.Mangin@acri-st.fr

**Keywords:** CubeSat attitude determination, TRIAD, Kalman filter, climate

## Abstract

Ultraviolet and infrared sensors at high quantum efficiency on-board a small satellite (UVSQ-SAT) is a CubeSat dedicated to the observation of the Earth and the Sun. This satellite has been in orbit since January 2021. It measures the Earth’s outgoing shortwave and longwave radiations. The satellite does not have an active pointing system. To improve the accuracy of the Earth’s radiative measurements and to resolve spatio-temporal fluctuations as much as possible, it is necessary to have a good knowledge of the attitude of the UVSQ-SAT CubeSat. The attitude determination of small satellites remains a challenge, and UVSQ-SAT represents a real and unique example to date for testing and validating different methods to improve the in-orbit attitude determination of a CubeSat. This paper presents the flight results of the UVSQ-SAT’s attitude determination. The Tri-Axial Attitude Determination (TRIAD) method was used, which represents one of the simplest solutions to the spacecraft attitude determination problem. Another method based on the Multiplicative Extended Kalman Filter (MEKF) was used to improve the results obtained with the TRIAD method. In sunlight, the CubeSat attitude is determined at an accuracy better than 3${}^{{}^{\circ}}$ (at one σ) for both methods. During eclipses, the accuracy of the TRIAD method is 14${}^{{}^{\circ}}$, while it reaches 10${}^{{}^{\circ}}$ (at one σ) for the recursive MEKF method. Many future satellites could benefit from these studies in order to validate methods and configurations before launch.

## 1. Introduction

Ultraviolet and infrared sensors at high quantum efficiency on-board a small satellite (UVSQ-SAT) is a scientific and technological demonstrator dedicated to the observation of essential climate variables [1]. UVSQ-SAT was launched into a Sun-synchronous orbit by the LATMOS with the Falcon 9 rocket on 24 January 2021. After a commissioning phase, the routine phase started on 13 March 2021. Since then, the CubeSat has been fully functional, and first results have been published [2]. The methods used by [1] to obtain these results (maps of the solar radiation reflected by the Earth and of the outgoing longwave radiation at the top of the atmosphere) do not take into account the attitude of the UVSQ-SAT CubeSat. To improve the accuracy of the results, it is highly recommended to have an excellent knowledge of the attitude of the UVSQ-SAT CubeSat. This would allow researchers to obtain the Earth’s incident flux on each UVSQ-SAT face, given that the CubeSat has Earth radiative sensors and photodiodes on all its faces [1].

To determine the attitude of the UVSQ-SAT CubeSat, two methods are used: the Tri-Axial Attitude Determination (TRIAD) method and the Multiplicative Extended Kalman Filter (MEKF) method. TRIAD is a basic method, which is implemented by considering that the instruments’ measurements can be easily related to the information from models in an inertial frame of reference at the satellite’s location. Then, it is necessary to find the perfect rotation matrix to move from one reference frame to another. The MEKF method improves the results obtained with the TRIAD method. It aims to calibrate and correct the data from noise and inaccuracies.

The most commonly used methods in the literature are TRIAD and MEKF. Indeed, there are very few examples of results associated with CubeSat attitude determination in orbit. Table 1 presents a detailed background of the recent studies related to the problem of satellite attitude determination (AD). Simulations show that the restitution of the attitude can be better than 1${}^{{}^{\circ}}$. Moreover, the Radio Aurora Explorer satellites 3U CubeSat [3] demonstrated that it was possible to obtain knowledge of a satellite’s attitude with an accuracy better than 1${}^{{}^{\circ}}$ (sunlight) in orbit. Despite the small size of the CubeSats, it seems possible to accurately determine their attitude in orbit. This is mainly valid when the satellite is aimed at the Sun because several sensors (solar photodiodes, magnetometers, and gyroscopes) are used to perform this task successfully. During eclipses, this determination is more complex. CubeSat attitude determination is still a challenge as they are small, and they still do not have active attitude determination and control systems (ADCS). Furthermore, new miniaturized space-based payloads are becoming increasingly complex and require accurate knowledge of the satellite attitude. One of the objectives of the UVSQ-SAT mission is to obtain knowledge of the CubeSat attitude with an accuracy better than a few degrees in sunlight.

This manuscript presents two methods to determine the attitude of the UVSQ-SAT CubeSat. This is an important step in the implementation process of the scientific results of the UVSQ-SAT space-based mission. Section 2 describes the inputs from the satellite along with the models and geometrical considerations of the two methods. Section 3 presents the implementation of the two different methods and how they differ. Section 4 shows the results obtained from the different methods and how the MEKF method has improved the accuracy of the determination of the UVSQ-SAT CubeSat attitude of the TRIAD method. Finally, Section 5 is dedicated to the presentation of conclusions and perspectives.

## 2. UVSQ-SAT Attitude Determination Considerations

This section presents a description of the sensors of the UVSQ-SAT CubeSat, reference frames and attitude representation, and the theoretical approach of the method.

### 2.1. Sensors Description

**Figure 1:**
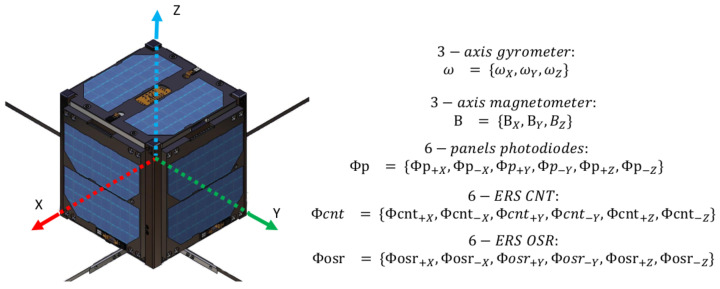
Spacecraft body frame of the UVSQ-SAT satellite.

The UVSQ-SAT satellite described by [1] is equipped with different subsystems and sensors, among which some are used to determine its attitude. Those instruments are defined in the spacecraft body frame (B). The different faces of the spacecraft are named after this reference frame. Two opposite faces correspond to one axis. This is shown in Figure 1. The instrumental reference frame is fixed with respect to the satellite. It undergoes only one constant rotation with respect to the satellite reference frame. To simplify this, we apply this rotation and consider the measurements in the spacecraft body frame. We do not mention the instrument frame in the following explanations.

The different inputs available to the algorithms are as follows:

- Three-axis angular velocities. The gyrometer measures the three-axis angular velocities in the sensor frame to the inertial reference frame (I), defined by $\omega_{g}={\left\{\omega_{X},\;\omega_{Y},\;\omega_{z}\right\}}_{B/I}$, as the calibrated measurement.
- Three-axis magnetic field. The magnetometer measures the magnetic field along its three axes in the instrument’s reference frame defined by $B={\left\{B_{X},\;B_{Y},\;B_{Z}\right\}}_{B}$, as the calibrated measurement.
- Six photodiodes in the visible domain. They measure solar and outgoing shortwave radiations in the 400–1100 nm wavelength range. They are defined as the calibrated fluxes $\Phi p=\{\Phi p_{+X},\;\Phi p_{-X},\;\Phi p_{+Y},\;\Phi p_{-Y},\;\Phi p_{+Z},\;\Phi p_{-Z}\}$. They are used as a Sun sensor.
- Six Earth radiative sensors (ERS) sensors with an Optical Solar Reflector (OSR). They aim to measure radiation between 0.2 and 3 μm. They are defined as $\Phi osr=\{\Phi osr_{+X},\;\Phi osr_{-X},\;\Phi osr_{+Y},\;\Phi osr_{-Y},\;\Phi osr_{+Z},\;\Phi osr_{-Z}\}$. Those sensors are used as an Earth sensor and aimed toward the nadir.
- Six ERS sensors with carbon nanotubes (CNT). Six sensors aim to measure total radiation between 0.2 and 100 μm. They are defined as $\Phi cnt=\{\Phi cnt_{+X},\;\Phi cnt_{-X},\;\Phi cnt_{+Y},\;\Phi cnt_{-Y},\;\Phi cnt_{+Z},\;\Phi cnt_{-Z}\}$. They are used as Earth sensors.

**Figure 2:**
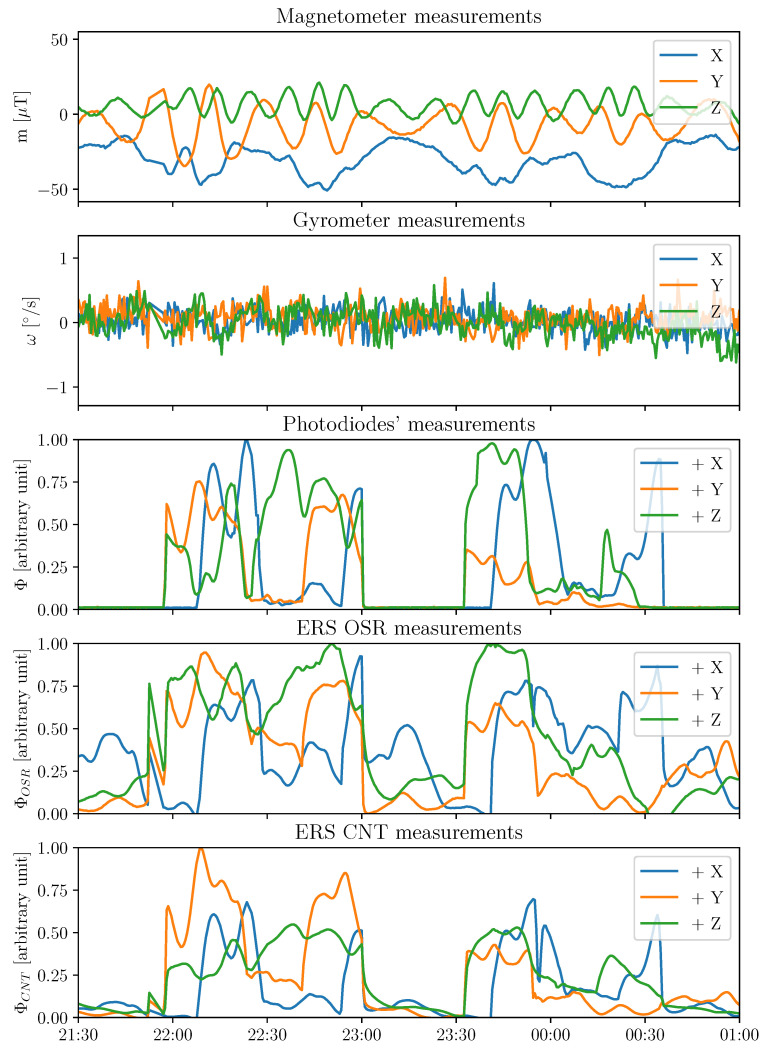
Time series of the measurements for two orbits on 26 March 2021 from the three-axis magnetometer, the three-axis gyrometer, the photodiodes, and the ERS sensors.

An example of the time series of the UVSQ-SAT inputs is given in Figure 2. Oscillations are present in the magnetometer and gyrometer measurements as the satellite rotates. For the photodiode measurements, sunlight and eclipses periods appear clearly. ERS sensors measure all Earth and solar radiative fluxes. Eclipses periods appear also clearly.

### 2.2. Orbital Reference Frames and Attitude Representation

#### 2.2.1. Orbital Reference Frames

**Figure 3:**
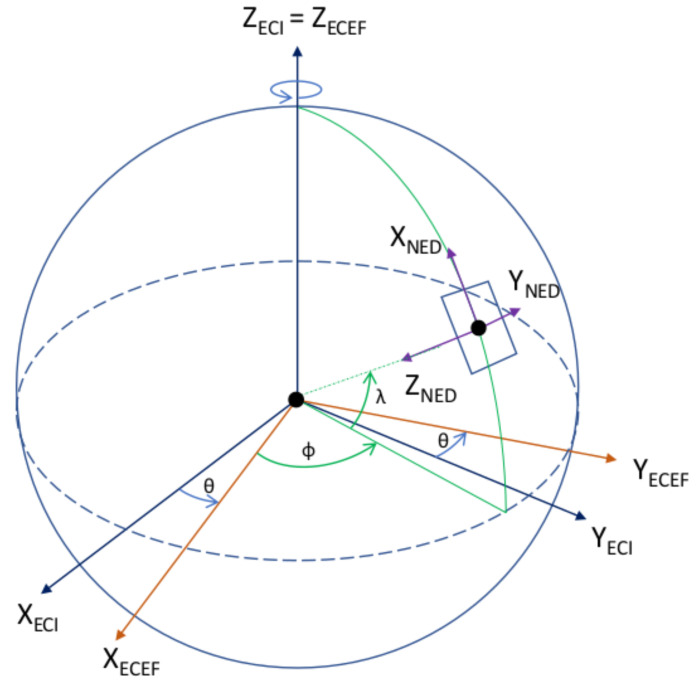
Earth-centered inertial (ECI), Earth-centered Earth-fixed (ECEF), and North East Down (NED) reference frames.

**Figure 4:**
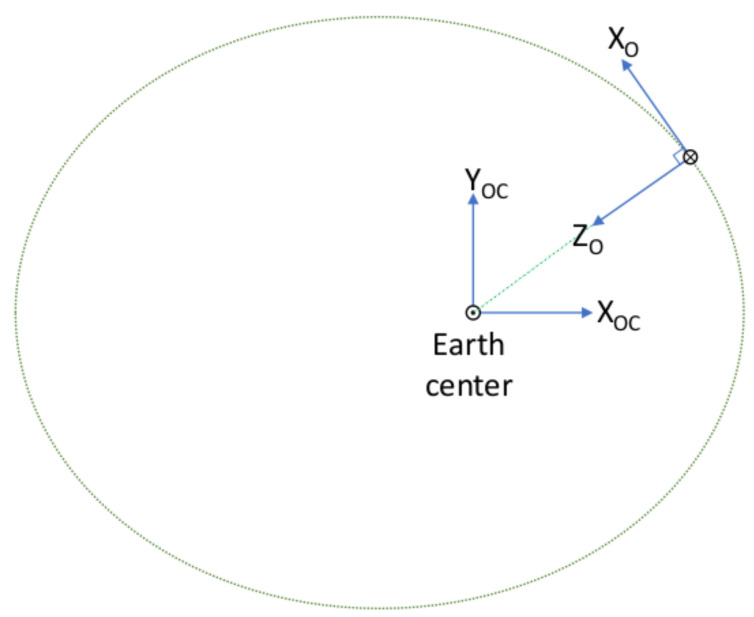
Earth centered orbit reference frame (OC) and orbit (O) reference frames.

**Figure 5:**
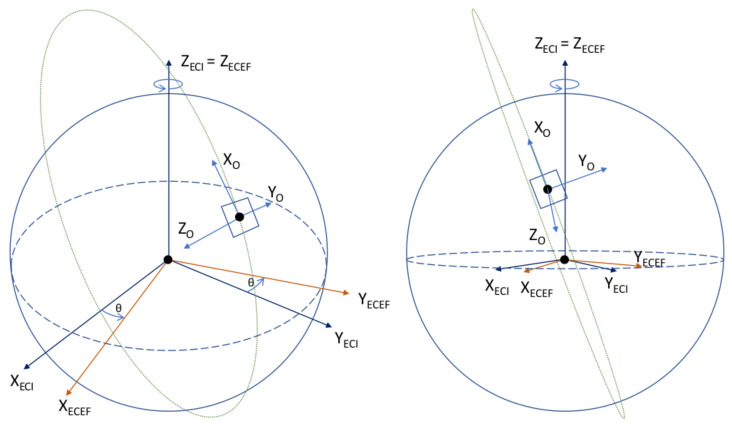
Earth-centered inertial (ECI), Earth-centered Earth-fixed (ECEF), and orbit (O) reference frames.

The following reference frames are required for the in-orbit attitude determination of the UVSQ-SAT CubeSat. They allow us to compute the satellite’s attitude with respect to one of those frames. The reference frames are shown in Figure 3, Figure 4 and Figure 5. We recall that the gyrometer measures an angular velocity in the body frame with respect to an inertial reference frame. Therefore, an essential reference frame defined here is the Earth-centered inertial (ECI) along with the Earth-centered orbit reference frame (OC). The reference frames are described as follows:

- Earth-centered inertial (ECI). The reference frame is defined in blue in Figure 3 with an origin at the Earth’s center of mass. The X-axis is defined as the vernal equinox axis at J2000, the intersection between the equatorial and the ecliptic planes. The Z-axis is defined as the Earth rotation axis at epoch J2000. Finally, the Y-axis is defined according to the previous directions to create an orthogonal basis.
- Earth-centered Earth-fixed (ECEF). The reference frame is defined in red in Figure 3 with its origin at the Earth’s center of mass. Its X-axis is defined at the intersection of the Greenwich prime meridian and the equator. Its Y-axis is the intersection of the equatorial plane and the 90${}^{{}^{\circ}}$ longitude. The Z-axis extends through the true north and south poles and coincides with the Earth’s rotation axis.
- North East Down (NED). Assuming a WGS84 ellipsoid model of the Earth, the NED, defined in purple in Figure 3, is a local reference frame that moves the body frame’s position in the ECEF. It is defined so that the X–Y plane is tangential to the surface of the ellipsoid at the given location in the ECEF. Given those conditions, the X-axis should point toward true North, the Z-axis toward the interior of the Earth, and the Y-axis will finalize the orthogonal basis.
- Earth-centered orbit reference frame (OC). The reference frame is defined in blue in Figure 4 and Figure 5 and centered at the Earth’s center, with the X-axis towards the perigee, the Y-axis along the semi-minor axis, and the Z-axis perpendicular to the orbital plane to complete the right-hand system. From the previous reference frame, it is thus necessary to define a local reference frame that will follow the satellite in its center. This reference frame is chosen for its logic with respect to the satellite motion as well as the possibility of taking into account the orbital velocity in order to correct the gyrometer of this frame.
- Orbit reference frame (O). The reference frame is defined such that its origin is located at the center of the spacecraft. The origin rotates relative to the ECI with an angular velocity of $\omega_{0}$. Its Z-axis points towards the center of the Earth. The X-axis is perpendicular to the previous axis in the spacecraft’s direction of motion. The Y-axis completes the right-hand system.

#### 2.2.2. Attitude Representation

**Figure 6:**
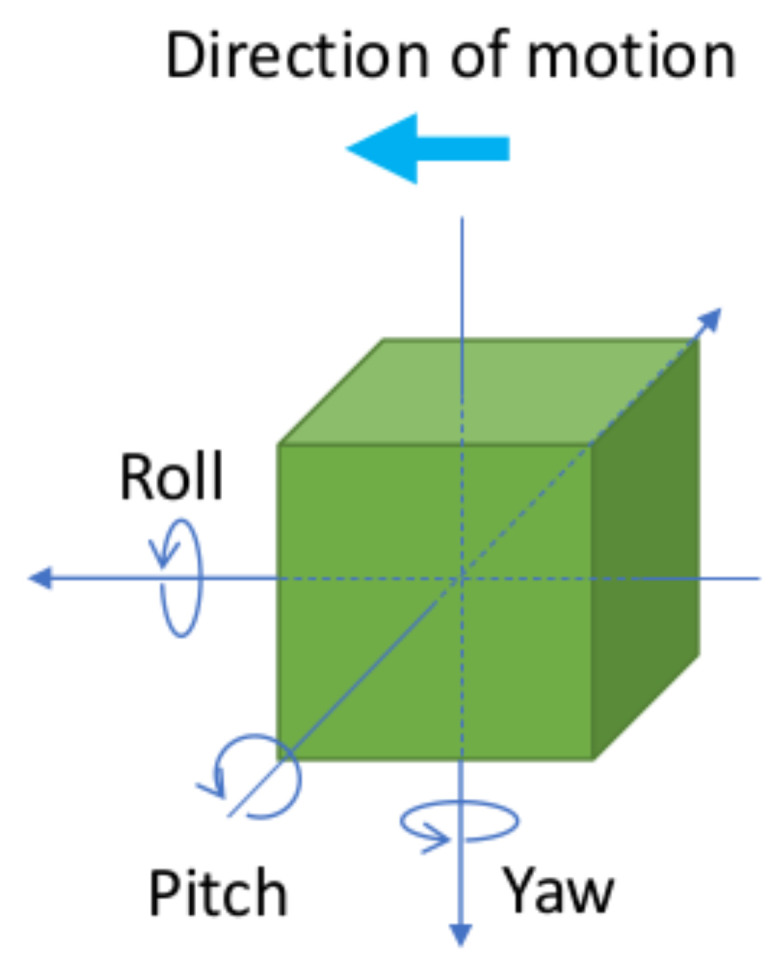
Euler angles defined for the satellite.

There are different ways to express the satellite attitude in its reference frame. The first basic representations are known as the Euler angles. The orientation of the body with respect to a reference frame is given by three Euler angles. Those angles define three successive rotations around different axes. Roll, pitch, and yaw angles are defined in Figure 6 and are called ϕ, θ, and ψ, respectively.

In order to avoid singularities that come with the choice of using Euler angles for attitude representation, we define the quaternion representation where a quaternion q is defined as

(1) $$q=q_{1}+q_{2}i+q_{3}j+q_{4}k=\left[\begin{array}{c} q_{1}\\ q_{2}\\ q_{3}\\ q_{4} \end{array}\right]$$

where $q_{1}$, $q_{2}$, $q_{3}$, and $q_{4}$ are real numbers with $\left\{1,i,j,\;\mathrm{and}\;k\right\}$ as a basis for a 4-dimensional vector space. $i^{2}=j^{2}=k^{2}=ijk=-1$ and as q≠0. The rotation resulting from the previous quaternion is characterized by its rotation angle α around its axis of coordinates $\left(r_{x},r_{y},r_{z}\right)$ defined in Equation (2a,b):

(2a) $$\alpha =2atan2\left(\sqrt{q_{2}^{2}+q_{3}^{2}+q_{4}^{2}},q_{1}\right)$$

(2b) $$\left(r_{x},r_{y},r_{z}\right)=\frac{\left(q_{2},q_{3},q_{4}\right)}{\sqrt{q_{2}^{2}+q_{3}^{2}+q_{4}^{2}}}$$

Then, we obtain Equation (3) as follows:

(3) $$\left[\begin{array}{c} q_{1}\\ q_{2}\\ q_{3}\\ q_{4} \end{array}\right]=\left[\begin{array}{c} \cos\frac{\alpha }{2}\\ r_{x}\sin\frac{\alpha }{2}\\ r_{y}\sin\frac{\alpha }{2}\\ r_{y}\sin\frac{\alpha }{2} \end{array}\right]$$

The quaternions and the Euler angles can be associated as (Equation (4))

(4) $$\begin{array}{c} q_{1}=\cos\left(\frac{\alpha }{2}\right)\\ q_{2}=\sin\left(\frac{\alpha }{2}\right)\cos\left(\alpha_{x}\right)\\ q_{3}=\sin\left(\frac{\alpha }{2}\right)\cos\left(\alpha_{y}\right)\\ q_{4}=\sin\left(\frac{\alpha }{2}\right)\cos\left(\alpha_{z}\right) \end{array}$$

where $\alpha_{x}$, $\alpha_{y}$, and $\alpha_{z}$ are the angles between the axis of rotation and the axes X, Y, and Z, respectively. From those two representations, we can create a third tool—the Direction Cosine Matrix (DCM)—that is used in the algorithm. We define the rotation matrix, also called the attitude matrix, which represents the rotation of the body in the body frame (B) with respect to a specified frame—for example, the orbital frame (O)—as follows in Equation (5a,b):

(5a) $$DCM_{O\overset{}{\to }B}\left(\phi ,\theta ,\psi \right)=\left[\begin{array}{ccc} 1 & 0 & 0\\ 0 & \cos\phi & \sin\phi \\ 0 & -\sin\phi & \cos\phi \end{array}\right]\left[\begin{array}{ccc} \cos\theta & 0 & -\sin\theta \\ 0 & 1 & 0\\ \sin\theta & 0 & \cos\theta \end{array}\right]\left[\begin{array}{ccc} \cos\psi & \sin\psi & 0\\ -\sin\psi & \cos\psi & 0\\ 0 & 0 & 1 \end{array}\right]$$

(5b) $$DCM_{O\overset{}{\to }B}\left(q\right)=\left[\begin{array}{ccc} 1-2s(q_{3}^{2}+q_{4}^{2}) & 2s(q_{2}q_{3}-q_{4}q_{1}) & 2s(q_{2}q_{4}+q_{3}q_{1})\\ 2s(q_{2}q_{3}+q_{4}q_{1}) & 1-2s(q_{2}^{2}+q_{4}^{2}) & 2s(q_{3}q_{4}-q_{2}q_{1})\\ 2s(q_{2}q_{4}-q_{3}q_{1}) & 2s(q_{3}q_{4}+q_{2}q_{1}) & 1-2s(q_{2}^{2}+q_{3}^{2}) \end{array}\right]$$

where *s* is the quaternion’s norm.

### 2.3. Theoretical Approach of the Method

The instruments and the reference frames are described in the previous section. It is now possible to present the different inputs required for attitude determination. Those inputs are the nadir direction, the Sun line-of-sight (LOS), the magnetic field vectors, and the gyrometer data. We suppose that those vectors are defined as follows (Equation (6a–c)):

(6a) $$\hat{S}\left(t\right)=S\left(t\right)+\eta_{S}\left(t\right)$$

(6b) $$\hat{N}\left(t\right)=N\left(t\right)+\eta_{N}\left(t\right)$$

(6c) $$\hat{B}\left(t\right)=B\left(t\right)+\eta_{B}\left(t\right)$$

where $\hat{S}\left(t\right)$, $\hat{N}\left(t\right)$, and $\hat{B}\left(t\right)$ are the Sun LOS, the nadir direction, and the magnetic field vectors retrieved from the instruments on-board the satellite, respectively. S(t), N(t) and B(t) are the true Sun LOS, the true nadir direction, and the true magnetic field vectors, respectively. $\eta_{S}\left(t\right)$, $\eta_{N}\left(t\right)$, and $\eta_{B}\left(t\right)$ are the three zero-mean Gaussian noises that we assume for the three vectors. According to Table 1, the use of a nadir direction in an eclipse to determine the satellite attitude is not common. In an eclipse, Table 1 shows that the most common idea is to rely only on magnetometer and gyrometer measurements. Indeed, we know that the gyrometer can be very noisy and inaccurate [21]. However, the UVSQ-SAT is equipped with the infrared sensors presented in Section 2. Thus, those infrared sensors will help to determine the nadir direction from the terrestrial infrared radiations.

The three-axis rate from the gyrometer is defined with $\hat{\omega }\left(t\right)$ as follows (Equation (7a,b)):

(7a) $$\hat{\omega }\left(t\right)=\omega \left(t\right)+\beta \left(t\right)+\eta_{\omega }\left(t\right)$$

(7b) $$\dot{\beta }\left(t\right)=\eta_{\beta }\left(t\right)$$

where ω(t) is the true rate, β(t) is the drift, and $\eta_{\omega }\left(t\right)$ and $\eta_{\beta }\left(t\right)$ are the zero-mean Gaussian noises.

As the measurements from the gyrometer are conducted with respect to an inertial reference frame, this is defined as (Equation 8)

(8) $$\omega_{B/I}=\omega_{B/\mathrm{OC}}+\omega_{\mathrm{OC}/I}$$

where $\omega_{\mathrm{OC}/I}$ is the rate from the body frame with respect to the inertial frame that is equal to $\omega_{0}$ the orbital angular velocity along the axis orthogonal to the orbital plane. $\omega_{0}$ is computed from the mean motion given at each time. This information is contained in a list of orbital elements for a given point in time called a two-line element set (retrieved by the NORAD). The angular velocity with respect to the orbital frame is computed at each time given the attitude matrix at that time with respect to the orbital frame in Equation (9).

(9) $$\omega_{B/O}=\omega_{B/I}-A_{O\overset{}{\to }B}\omega_{O/I}=\omega_{B/I}-A_{O\overset{}{\to }B}\omega_{0}$$

where $A_{O\overset{}{\to }B}$ is the rotation matrix from the local orbital frame to the body reference frame. The changes in the reference frame presented are directly related to the description of the reference frames in Section 2.2.1. The choice of the inertial reference frame to use has been meticulously made to facilitate the calculations in Equation (9).

## 3. Attitude Determination Methods

### 3.1. Tri-Axial Attitude Determination Method (TRIAD)

#### 3.1.1. Formulation of the Method

The TRIAD algorithm aims to determine the attitude of the CubeSat. The output of the algorithm is the rotation matrix from the orbit reference frame (O) to the body reference frame (B). This matrix is also called the attitude matrix. The calculations are done instantaneously using two known vectors in both of the reference frames. The inputs required to compute the attitude matrix are the Sun LOS and the magnetic field in the two reference frames. In the body reference frame, the vectors are determined from the UVSQ-SAT measurements. In the orbit reference frame, the inputs are computed from the International Geomagnetic Reference Field [22] and orbital models at the satellite’s location and time. We note that in an eclipse, we use nadir vectors instead of Sun LOS vectors. The nadir vector is defined as N in the body frame and $N_{o}$ in the local orbital frame. The method is described in sunlight in the Equations (10) and (11). For the eclipse periods, N and S would commute. Nevertheless, less accurate results are expected in an eclipse since it is more complicated to determine the nadir from the infrared sensors rather than the Sun LOS.

**Figure 7:**
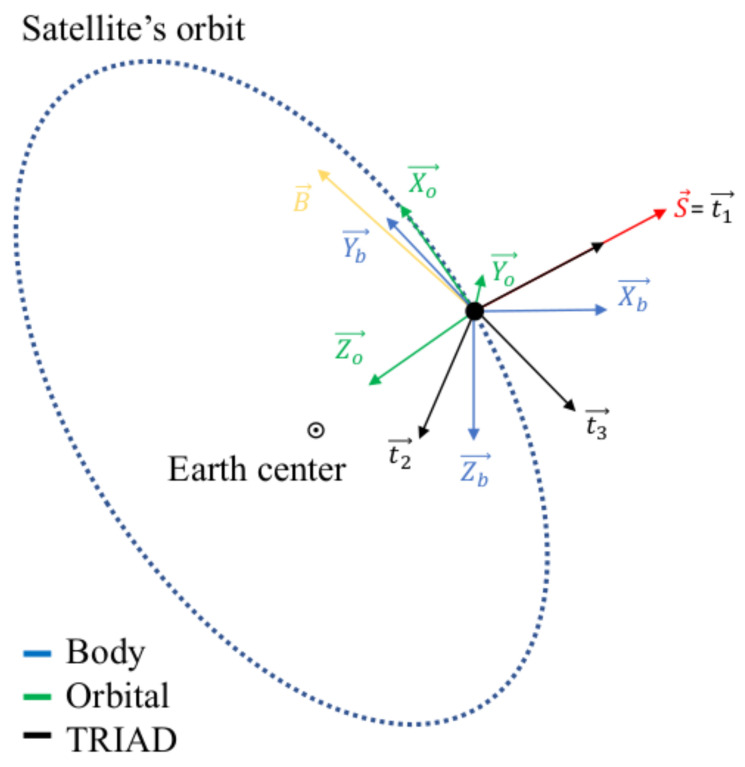
Reference frames for TRIAD method.

To compute the attitude from the TRIAD algorithm, we introduce a new reference frame based on the body and orbital reference frames. This new reference frame is called the TRIAD frame and was described in [23,24]. The TRIAD frame is meant to be an intermediary between the orbital and the body frame; therefore, it should be easily defined in each of those reference frames. This reference frame is based on the magnetic field and the Sun LOS vectors. Let us start by expressing the TRIAD frame in the body reference frame, which is described as $\left\{t_{1b},\;t_{2b},\;t_{3b}\right\}$ in Figure 7. Ideally, the most accurate vector should be used as the first axis. Usually, Sun sensors are more accurate than magnetometers. The Sun LOS is therefore chosen as the first direction. This axis is often called the anchor as it remains unchanged. Thus, the frame can be described in the body reference frame as (Equation (10))

(10) $$t_{1b}=\frac{S}{∥S∥},\;t_{2b}=\frac{S\times B}{∥S\times B∥},\;t_{3b}=t_{1b}\times t_{2b}$$

where S and B are the Sun LOS and magnetic field vector in the body frame retrieved from the instruments on-board the spacecraft, and the TRIAD’s basis can be expressed as $\left\{t_{1o},\;t_{2o},\;t_{3o}\right\}$ in the orbital frame, in Equation (11):

(11) $$t_{1o}=\frac{S_{o}}{∥S_{o}∥},\;t_{2o}=\frac{S_{o}\times B_{o}}{∥S_{o}\times B_{o}∥},\;t_{3o}=t_{1o}\times t_{2o}$$

where $S_{o}$ and $B_{o}$ are the Sun LOS and magnetic field vectors in the orbital frame computed from models. Therefore, it is rather simple to recover the transfer matrix from the orbital to body frame via the TRIAD frame. The rotation matrix can be written as in Equation (12):

(12) $$R_{TRIAD,O\overset{}{\to }B}=\left[t_{1o},\;t_{2o},\;t_{3o}\right]{\left[t_{1b},\;t_{2b},\;t_{3b}\right]}^{T}$$

#### 3.1.2. Optimized TRIAD

One of the limitations of the TRIAD method presented in Section 3.1.1 is the dependence on the choice of the first direction. This direction, called the anchor, remains untouched through the TRIAD process. However, in reality, neither of the two vectors used are perfectly aligned with the model. Therefore, in [25], the authors proposed to improve the method by taking the relative accuracy of the two measurements into account. The idea is to weight the two attitude matrices corresponding to the choice of using either of the two vectors as the first direction. This algorithm is called the optimized TRIAD algorithm. We define $\sigma_{S}$ and $\sigma_{B}$ as the standard deviations of the error of the LOS vector and the magnetic field vector, respectively. The attitude matrices $A_{S}$ and $A_{B}$ computed via the TRIAD method are for using the Sun LOS vector and magnetic field vector, respectively, as the first direction of the TRIAD frame. The weighting process is done as follows (Equation (13)).

(13) $$A^{*}=\frac{\sigma_{S}^{2}}{\sigma_{S}^{2}+\sigma_{B}^{2}}A_{S}+\frac{\sigma_{B}^{2}}{\sigma_{S}^{2}+\sigma_{B}^{2}}A_{B}$$

In order to obtain an attitude matrix, the resulting matrix must be orthogonal; therefore, the final attitude matrix is obtained in Equation (14). According to [26], one orthogonalization cycle is needed as $A^{*}$ is close to being orthogonal.

(14) $$A=\frac{1}{2}\left[A^{*}+{\left(A^{*-1}\right)}^{T}\right]$$

Coarse Sun sensors are obviously not as accurate as Sun sensors. Therefore, it is quite legitimate to take the relative uncertainties of the two components into account via the presented method.

As for the simple TRIAD algorithm, no noise correction is applied. In case of large noise or, for example, high variability of the magnetic field, the computed attitude would not be accurate.

### 3.2. Multiplicative Extended Kalman Method

The MEKF method aims to improve the attitude determination accuracy by correcting instrument noise and calibrating the gyrometer in real-time. The Kalman filter was described by Swerling in 1958 or Kalman [27] and Kalman and Bucy [28]. The principle is based on a two-step method that aims to correct noises and instrument uncertainties. The state variables describe the system at each time. These variables provide information on the corrections to be made to the instruments as well as the orientation of the satellite. They are first estimated and then corrected based on the observation from the instruments. Although standard Kalman filters are truly efficient for linear systems, they cannot be accurate for non-linear systems. Therefore, in our case, we use an Extended Kalman Filter (EKF). This algorithm is a linearized Kalman filter at the point of reference using the Taylor series expansions principle.

#### 3.2.1. Formulation

At each iteration, MEKF [29] uses the quaternion as the attitude representation and the state vector δϑ for the representation of the attitude errors. The true quaternion can then be defined as follows (Equation (15)):

(15) $$q^{\mathrm{true}}=\delta q\left(\delta \vartheta \right)⊗\hat{q}$$

where $q^{\mathrm{true}}$ is the true quaternion that represents the real orientation of the object that is defined from a product. $\hat{q}$ is the estimate quaternion giving an estimate of the object orientation. δq(δϑ) is the error quaternion defined by δϑ, with the three components representing the attitude error. ⊗ is the quaternion product symbol first used in [30] and defined in [29] as follows (Equation (16)):

(16) $$\bar{q}⊗q=\left[\begin{array}{c} q_{4}{\bar{q}}_{1:3}+{\bar{q}}_{4}q_{1:3}+{\bar{q}}_{1:3}\times q_{1:3}\\ {\bar{q}}_{4}q_{4}-{\bar{q}}_{1:3}.q_{1:3} \end{array}\right]$$

where $\hat{x}$ represents the estimate of the quantity *x* (for example, the state vector). ${\hat{x}}^{+}$ represents the updated quantity of ${\hat{x}}^{-}$, before being updated. The local attitude error is the true linearized variable of interest to compute the attitude at each iteration. However, the gyrometer which is required to predict the motion of the satellite has several calibration parameters that need to be computed, such as the misalignments, scale factors, and time-dependent drift biases. Therefore, those quantities must be computed at each iteration. So, they must be considered as state variables.

#### 3.2.2. Initialization

The state vector as mentioned previously is computed as (Equation (17))

(17) $${\hat{x}}_{0}=\left[\delta {\hat{\vartheta }}_{0}^{T}{\hat{\beta }}_{0}^{T}{\hat{s}}_{0}^{T}{\hat{k}}_{U0}^{T}{\hat{k}}_{L0}^{T}\right]$$

where ${\hat{\beta }}_{0}$ is the initial gyro drift biases. ${\hat{s}}_{0}$ is the initial gyrometer scale factor. ${\hat{k}}_{U0}$ and ${\hat{k}}_{L0}$ are the initial misalignments. $P_{0}$ is the initial covariance matrix defined from the predicted instrument uncertainties. $\delta {\hat{\vartheta }}_{0}=0_{3}$ is the initial attitude error for ${\hat{q}}_{0}$ (initial quaternion).

#### 3.2.3. Gain

The Kalman gain is used to give different weights to the measurements and the current estimate of the state. This is the weight assigned to the prediction or the observation and is defined by $K_{k}$ as (Equation (18))

(18) $$K_{k}=P_{k}^{-}H_{k}^{T}\left({\hat{x}}_{k}^{-}\right){\left[H_{k}\left({\hat{x}}_{k}^{-}\right)P_{k}^{-}H_{k}^{T}\left({\hat{x}}_{k}^{-}\right)+R_{k}\right]}^{-1}$$

where $H_{k}\left(x_{k}^{-}\right)$ is the observation model at time $t_{k}$. $R_{k}$ is the measurement-error covariance matrix at $t_{k}$. $P_{k}$ is the state error covariance at $t_{k}$. $H_{k}\left(x_{k}^{-}\right)$ is the observation matrix and is defined by (Equation 19):

(19) $$H_{k}\left(x_{k}^{-}\right)=\left[\begin{array}{c} A\left({\hat{q}}^{-}\right)B_{o}\times \;|\;0_{3\times 12}\\ A\left({\hat{q}}^{-}\right)S_{o}\times \;|\;0_{3\times 12}\\ A\left({\hat{q}}^{-}\right)N_{o}\times \;|\;0_{3\times 12} \end{array}\right]$$

The operator × is such that (Equation (20))

(20) $$x_{1:3}\times =\left[\begin{array}{ccc} 0 & -x_{3} & x_{2}\\ x_{3} & 0 & -x_{1}\\ -x_{2} & x_{1} & 0 \end{array}\right]$$

where A(q) is the attitude matrix in Equation (21):

(21) $$A\left(q\right)={∥q∥}^{-2}\left(\left(q_{4}^{2}-{∥q_{1:3}∥}^{2}\right)I+2q_{1:3}q_{1:3}^{T}-2q_{4}\left[q_{1:3}\times \right]\right))$$

#### 3.2.4. Update

This subsection aims to compute the post-update of the different variables at time $t_{k}$. The covariance matrix *P* can then be post-updated in Equation (22):

(22) $$P_{k}^{+}=\left[I-K_{k}H_{k}\left({\hat{x}}_{k}^{-}\right)\right]P_{k}^{-}$$

At this phase, a reset is applied to the pre-estimate of the error angle in Equation (23):

(23) $$\delta {\hat{\vartheta }}_{k}^{-}=0_{3}$$

It is then possible to update the state vector in Equation (24):

(24) $${\hat{x}}_{k}^{+}={\hat{x}}_{k}^{-}+K_{k}\left[y_{k}-h_{k}\left({\hat{x}}_{k}^{-}\right)\right]$$

where ${\hat{x}}_{k}=\left[\delta {\hat{\vartheta }}_{k}^{T}\;{\hat{\beta }}_{k}^{T}\;{\hat{s}}_{k}^{T}\;{\hat{k}}_{U_{k}}^{T}\;{\hat{k}}_{L_{k}}^{T}\right]$. $h_{k}\left({\hat{x}}_{k}^{-}\right)$ is the estimated observation that is given in Equation (25). The measurements are given in Equation (26).

(25) $$h_{k}\left({\hat{x}}_{k}^{-}\right)={\left[\begin{array}{c} A\left({\hat{q}}^{-}\right)B_{o}\\ A\left({\hat{q}}^{-}\right)S_{o}\\ A\left({\hat{q}}^{-}\right)N_{o} \end{array}\right]}_{t_{k}}$$

(26) $$y_{k}={\left[\begin{array}{c} B_{}\\ S_{}\\ N_{} \end{array}\right]}_{t_{k}}$$

The quaternion’s update is performed through two steps that aim to compute the quaternion corresponding to the error estimate in Equation (27a) and to preserve the unit quaternion norm in Equation (27b).

(27a) $${\hat{q}}^{*}={\hat{q}}_{k}^{-}+\frac{1}{2}\Xi \left({\hat{q}}_{k}^{-}\right)\delta {\hat{\vartheta }}_{k}^{+}$$

(27b) $${\hat{q}}_{k}^{+}=\frac{q^{*}}{∥q^{*}∥}$$

where $\delta {\hat{\vartheta }}_{k}^{+}$ corresponds to the first three components of the state vector ${\hat{x}}_{k}^{+}$ and the Ξ(q) matrix is defined in Equation (28):

(28) $$\Xi \left(q\right)=\left[\begin{array}{c} q_{4}I_{3}+\left[q_{1:3}\times \right]\\ -q_{1:3}^{T} \end{array}\right]$$

#### 3.2.5. Propagation

The propagation part aims to propagate the expected values and covariance at time $t_{k+1}$. First of all, we compute the estimate of the gyrometer components corrected based on the factors, drifts, and misalignments obtained from the previous step as (Equation (29))

(29) $${\hat{\omega }}^{+}\left(t\right)=\left[I_{3}-\hat{S}\left(t\right)\right]\left[{\hat{\omega }}^{-}\left(t\right)-\hat{\beta }\left(t\right)\right]$$

where *S* characterizes the gyrometer biases and misalignments and is defined as (Equation (30)):

(30) $$S=\left[\begin{array}{ccc} s_{1} & k_{U1} & k_{U2}\\ k_{L1} & s_{2} & k_{U3}\\ k_{L2} & k_{L3} & s_{3} \end{array}\right]$$

Based on the knowledge of the updated angular rate and quaternion at time $t_{k}$, the quaternion’s estimate at time $t_{k+1}$ is defined in Equation (31) as

(31) $${\hat{q}}_{k+1}^{-}=\bar{\Theta }\left({\hat{\omega }}_{k}^{+}\right){\hat{q}}_{k}^{+}$$

where $\bar{\Theta }\left({\hat{\omega }}_{k}^{+}\right)$ is defined as (Equation (32))

(32) $$\bar{\Theta }\left({\hat{\omega }}_{k}^{+}\right)=\left[\begin{array}{cc} \cos\left(\frac{1}{2}∥{\hat{\omega }}_{k}^{+}∥\Delta t\right)I_{3}-\left[{\hat{\Psi }}_{k}^{+}\times \right] & {\hat{\Psi }}_{k}^{+}\\ -{\hat{\Psi }}_{k}^{+} & \cos\left(\frac{1}{2}∥{\hat{\omega }}_{k}^{+}∥\Delta t\right) \end{array}\right]$$

where Δt is the duration between the two considered epochs. ${\hat{\Psi }}_{k}^{+}$ is computed in Equation (33) as

(33) $${\hat{\Psi }}_{k}^{+}=\frac{\sin\left(\frac{1}{2}∥{\hat{\omega }}_{k}^{+}∥\Delta t\right){\hat{\omega }}_{k}^{+}}{∥{\hat{\omega }}_{k}^{+}∥}$$

The covariance matrix *P* estimate at $t_{k+1}$ is such that (Equation (34))

(34) $$P_{k+1}^{-}=\Phi_{k}P_{k}^{+}\Phi_{k}^{T}+\Gamma_{k}Q_{k}\Gamma_{k}^{T}$$

where $Q_{k}$ is defined as in Equations (35)–(37) at the initialization and during the iterations as

(35) $$Q\left(t\right)=block\;\;diagonal(\left[\sigma_{v}^{2}I_{3}\;\;\sigma_{u}^{2}I_{3}\;\;\sigma_{s}^{2}I_{3}\;\;\sigma_{U}^{2}I_{3}\;\;\sigma_{L}^{2}I_{3}\right])$$

(36) $$Q_{k}=\Delta t\;G\;Q\;G^{T}$$

(37) $$G\left(t\right)=\left[\begin{array}{cc} -(I_{3}-\hat{S}) & 0_{3\times 12}\\ 0_{12\times 3} & I_{12} \end{array}\right]$$

where $\Phi_{k}$ is the state transition matrix that is computed as follows (Equations (38)–(40b)) for a simple first-order approximation:

(38) $$\Phi_{k}=I_{15}+\Delta tF\left(t_{k}\right)$$

(39) $$F\left(t\right)=\left[\begin{array}{ccccc} -[\hat{\omega }\left(t\right)\times ] & -(I_{3}-\hat{S}) & diag(\omega -\hat{\beta }) & -\hat{U} & -\hat{L}\\ 0_{12\times 3} & 0_{12\times 3} & 0_{12\times 3} & 0_{12\times 3} & 0_{12\times 3} \end{array}\right]$$

(40a) $$\hat{U}=\left[\begin{array}{ccc} \omega_{2}-{\hat{\beta }}_{2} & \omega_{3}-{\hat{\beta }}_{3} & 0\\ 0 & 0 & \omega_{3}-{\hat{\beta }}_{3}\\ 0 & 0 & 0 \end{array}\right]$$

(40b) $$\hat{L}=\left[\begin{array}{ccc} 0 & 0 & 0\\ \omega_{1}-{\hat{\beta }}_{1} & 0 & 0\\ 0 & \omega_{1}-{\hat{\beta }}_{1} & \omega_{2}-{\hat{\beta }}_{2} \end{array}\right]$$

These calculation steps are applied to the inputs for each time step. In the case where the uncertainties related to the dynamics model are not very important, the corrections related to the gyro will converge at least in the short term. Nevertheless, they could evolve with the aging of the instrument and the flight conditions; thus, real-time calibration is legitimate.

## 4. Results

Once the algorithm is implemented, it is possible to calculate the satellite attitude for each measurement acquisition. The objective of calculating this attitude is required to compute the incident flux for each face of the CubeSat, as presented in Section 1. This means that the measured fluxes are corrected from their angle to the observed source. Therefore, the flux corrections are directly related to the Sun LOS and nadir vectors in the spacecraft body frame.

This section presents the two unit vectors in the satellite’s body reference frame for both TRIAD and MEKF methods. The vectors are represented as a function of time according to each of their components in the satellite reference frame.

For example, if the X component of the Sun LOS is equal to 1, the satellite’s +X face is facing the Sun, the normal to the face coincides with the Sun LOS vector, and this would be the case for the −X face if the Sun LOS X component was −1. In the following studies, only data from the UVSQ-SAT satellite in orbit are used to test the TRIAD and MEKF methods.

### 4.1. Results with TRIAD Method

**Figure 8:**
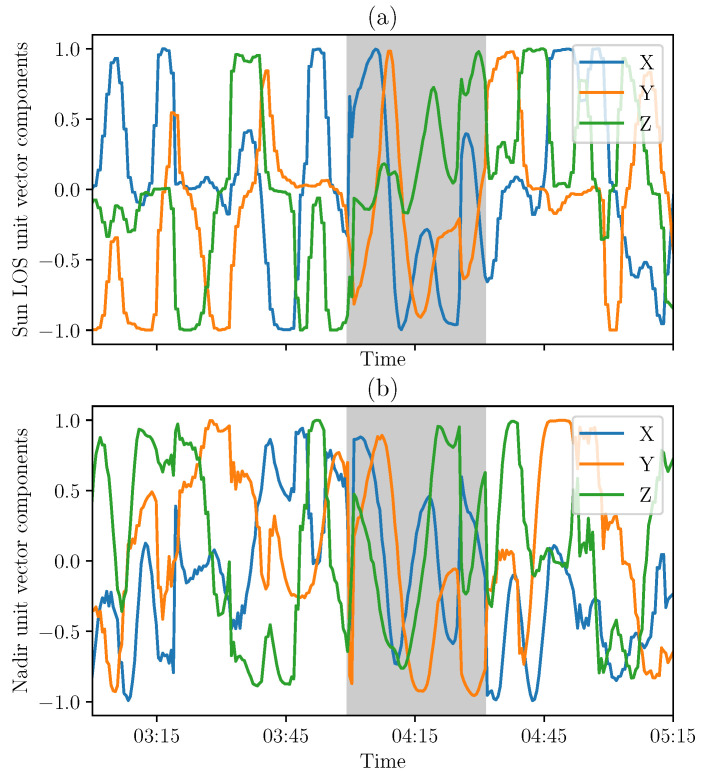
First lights of the Sun LOS (**a**) and nadir (**b**) components in the satellite body frame based on TRIAD method on 2 March 2021 (representative period in the case of measurements at 30 s intervals almost continuously, with eclipse phase in gray).

The TRIAD method (Section 3.1) provides the CubeSat attitude matrix. The Sun LOS in the body frame is computed from the model and the attitude matrix. The nadir direction in the body frame is obtained from the transformation of the nadir vector in the orbital frame (defined as the third axis of the reference frame basis). Those two vectors are represented in Figure 8.

### 4.2. Results with MEKF Method

**Figure 9:**
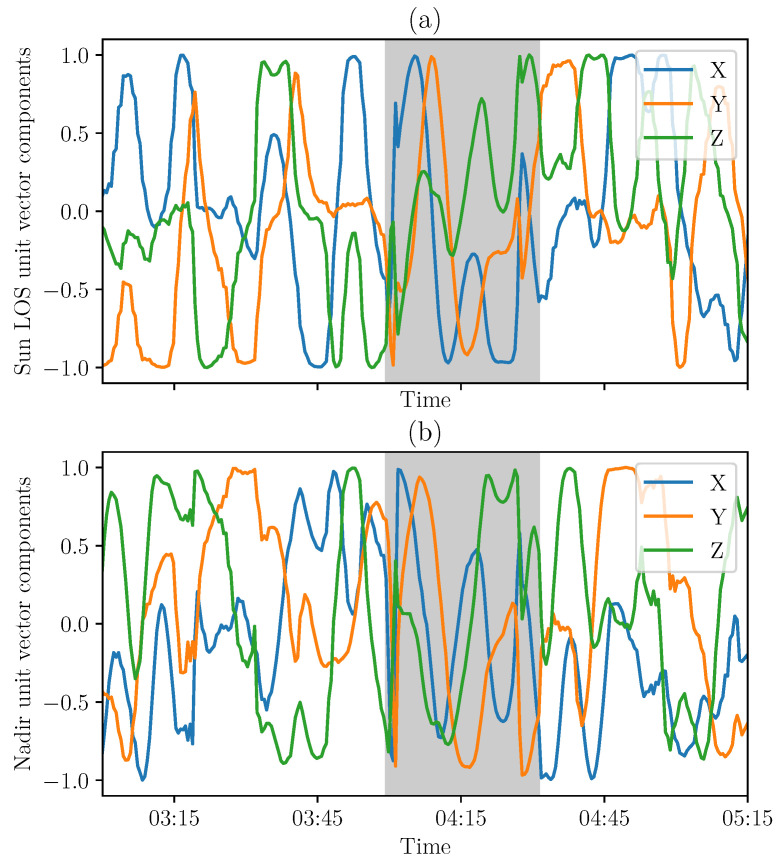
First lights of the Sun LOS (**a**) and nadir (**b**) components in the satellite body frame based on MEKF on 2 March 2021 (representative period in the case of measurements at 30 s intervals almost continuously with eclipse phase in gray).

The second method, described in Section 3.2, should correct the signals from noise and gyrometer biases. The Sun LOS and nadir directions are computed in the body reference frame from the attitude matrix. Their components in this frame are shown in Figure 9.

### 4.3. Discussion and Perspectives

**Figure 10:**
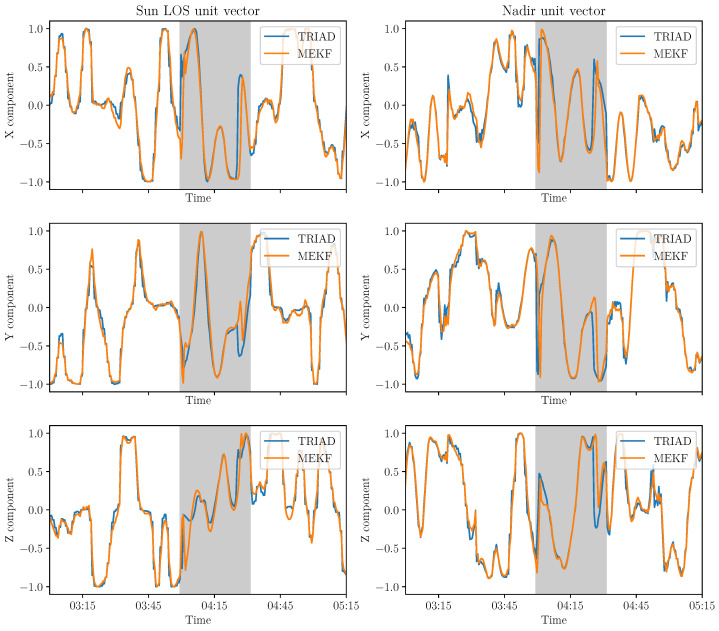
First lights of the Sun LOS and nadir coordinates in the satellite body frame based on MEKF and TRIAD on 2 March 2021 (representative period in the case of measurements at 30 s intervals almost continuously with eclipse phase in gray).

The TRIAD and MEKF methods were applied to in-orbit data. The objective is to compare the two methods. To visualize the improvement expected from the MEKF method, it can be wise to superpose the results from the different methods in Figure 10. Three indicators to compare the methods are described in this part. The first indicator is based on the variations of the attitude itself while the second one is derived from the measurements of a sensor that was not used as an input by the two methods. The last indicator is the uncertainty of each method. The satellite attitude computed with the Kalman filter is continuous, which corresponds to a realistic case in orbit. Discontinuities mostly come from noise in the measurement process. It is therefore relevant to quantify the fast changes in attitude. This can be done studying the gradient of the Sun LOS or nadir components. Studying the standard deviation of the gradient is representative of the short variations that could be due to the attitude determination error. The average of the standard deviations of the Sun LOS gradients is equal to 5.17 × 10${}^{-3}$$s^{-1}$ for the TRIAD method compared to 4.1 × 10${}^{-3}$$s^{-1}$ for the MEKF method (UVSQ-SAT data on the 2 and 3 February 2021). Therefore, the MEKF method seems to represent the satellite attitude variations better based on the previous indicator as it reduces discontinuities thanks to its smoothing asset.

A more accurate analysis can be established based on the measurements of a sensor not used in the attitude determination process. UV sensors (UVSs) with a narrow field of view (FOV) are used to compare the results based on the two methods. Those sensors are primarily sensitive to solar radiation. Therefore, the sensors should detect when the Sun appears in the FOV. It is therefore a great indicator to evaluate the precision and accuracy of both methods. This allows us to determine which method is the most adequate to find the orientation of the satellite (in the majority of cases). It is important to note that this study has limitations as it is realized only in the phases of sunlight and in some configurations. The reference cases are related to direct solar observations.

The accuracy and precision values are computed for each method. The accuracy value is defined in Equation (41a) as the proximity of a measured value to a real value. The value of precision in Equation (41b) refers to the proximity of two or more measurements to each other. This allows us to monitor the presence of a bias in the attitude determination.

(41a) $$Accuracy=\frac{TP+TN}{TP+FP+FN+TN}$$

(41b) $$Precision=\frac{TP}{TP+FP}$$

where *TP*, *FN*, *FP*, and *TN* are defined in Table 2.

**Figure 11:**
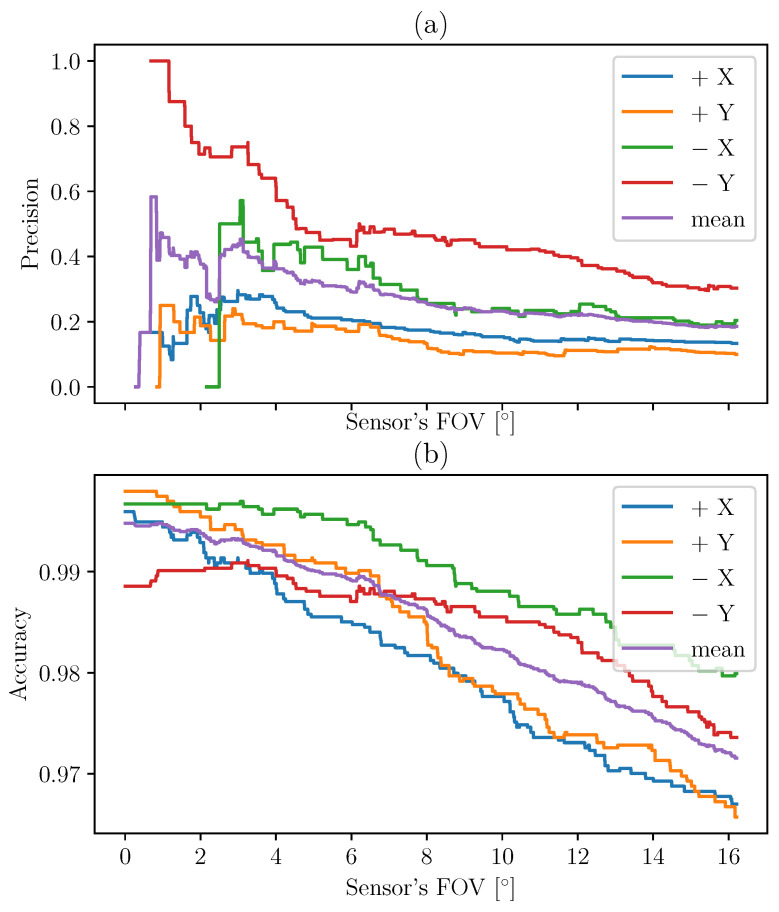
Precision (**a**) and accuracy (**b**) of the TRIAD method based on the UVS indicator.

**Figure 12:**
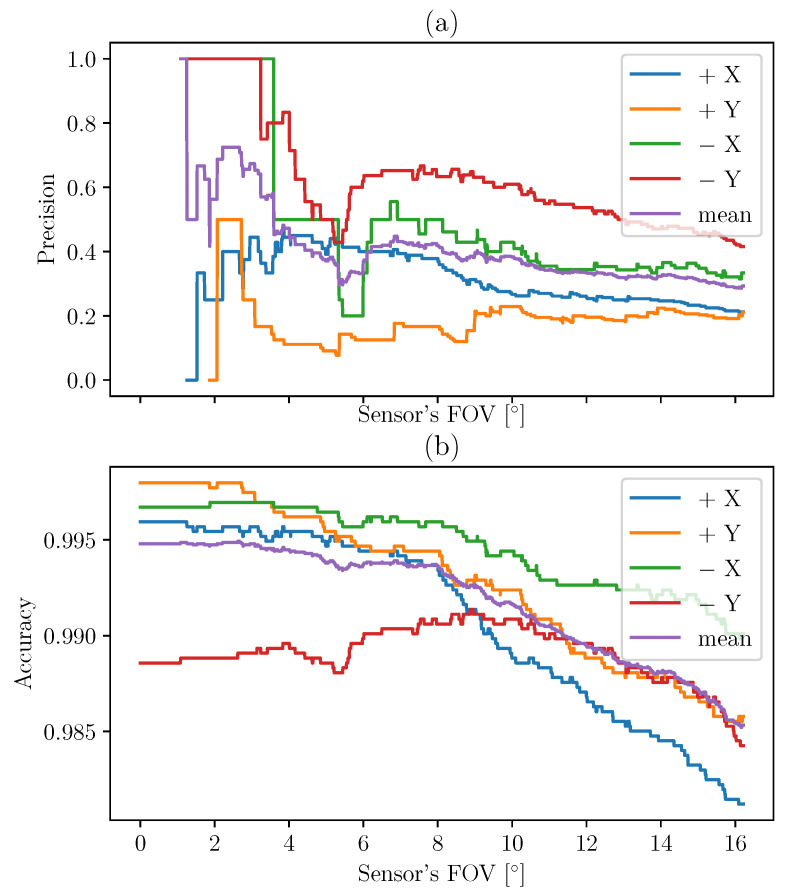
Precision (**a**) and accuracy (**b**) of the MEKF method based on the UVS indicator.

These values are calculated in the case of the TRIAD method in Figure 11 and then for MEKF in Figure 12.

The indicators are plotted according to different FOV. Indeed, the FOV of the instrument can be different from the theoretical FOV due to uncertainties of measurements and misalignments. This is the reason why we choose to calculate the accuracy and precision for different values of angle threshold (theoretical FOV). When the angle between the normal to the sensor and the Sun LOS determined by each method is below a certain threshold (FOV), it is verified that the UVS sensor receives a signal corresponding to the incident solar flux. This is an indicator that the considered face is facing the Sun. Using large numbers of values corresponding to large numbers of different configurations helps us to quantify the ability of the methods to recover the Sun LOS in the body frame.

**Figure 13:**
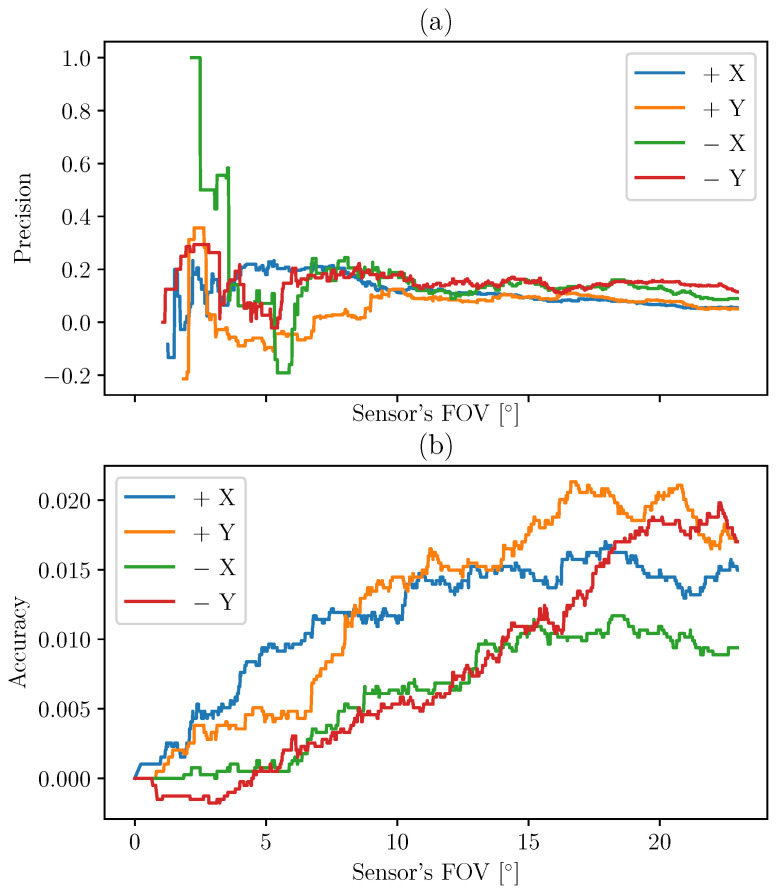
Difference between the precision (**a**) and accuracy (**b**) of the MEKF and the TRIAD methods based on the UVS indicator (positive means that MEKF has better performance compared to TRIAD).

The methods can be compared by computing the difference between the indicators for MEKF and TRIAD. The results are presented in Figure 13. The MEKF method allows us to increase the precision by about 20% and the accuracy by a few percent.

**Figure 14:**
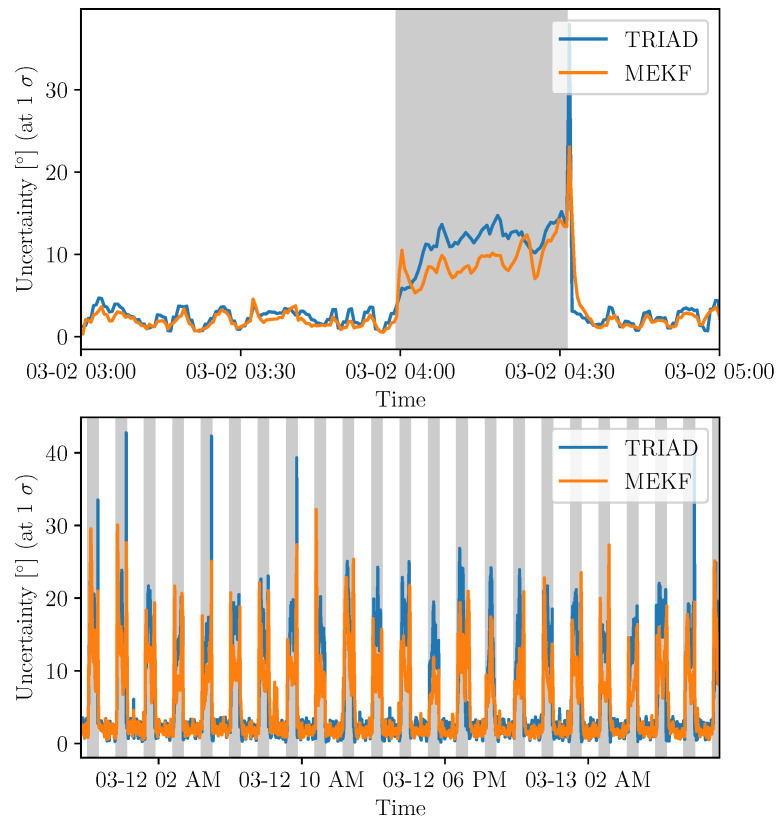
Uncertainties estimated from the Monte Carlo method for TRIAD and MEKF methods for continuous sampling on 2 March 2021 (**top**) and from 11–13 March 2021 (**bottom**) with eclipse phases in gray.

The last indicator used to compare the methods is the absolute uncertainty of the two methods developed above. To do this, we choose to use the Monte Carlo method to perform the propagation of uncertainties. The uncertainties of the instruments are quantified from the specifications and empirically. They are represented as a Gaussian probability density function. We then seek to quantify the uncertainty propagated on the angle to the Sun and the angle to the nadir as the flux computation depends on those parameters. The uncertainty is estimated in two different cases. The plot at the top of Figure 14 shows the evolution of the uncertainty for TRIAD and MEKF methods in the case of the data received almost continuously over the whole selected period. The sampling rate is thus almost constant and regular. This represents the ideal case where all the data are retrieved, and the only limitation is the sampling rate. The plot at the bottom of Figure 14 represents the same evolution for a longer period equivalent to more than 22 orbits including phases with missing data. It is thus possible to obtain the restitution of attitude with a 3${}^{{}^{\circ}}$ uncertainty (at 1 σ) in sunlight for both methods. In eclipse, for long periods of time (including missing data), the uncertainty reaches 14${}^{{}^{\circ}}$ for TRIAD while it converges to about 10${}^{{}^{\circ}}$ for the MEKF method. In general, we see an improvement of about 4${}^{{}^{\circ}}$. Eclipse phases appear in gray and sunlight in white. The determination of the uncertainty in time is pertinent as the attitude will be time-dependent for the MEKF method. This is apparent in Figure 14 as the uncertainty for the MEKF method converges with time. We also see that sunlight and eclipse phases have totally different results in terms of uncertainty as the data provided come from different instruments.

The accuracy of the MEKF method can be compared to validation with in-orbit data from [3]. It appears that the uncertainty is much lower for the Radio Aurora Explorer satellites with a 1 Hz sampling rate (below 1${}^{{}^{\circ}}$). A 1 Hz sampling rate was not enough to capture the spacecraft motion, and a slowing down of the satellite rotation was therefore undertaken and allowed to improve the results. In our case, the sampling period of 30 s does not allow us to reach such accuracy. This is nevertheless important to note as feedback.

It is possible to estimate the uncertainties on the incident flux from the uncertainties on the angles to the source. Indeed, fluxes are corrected using the cosine of the angle for the Sun and the view factor in the case of terrestrial flux. Those uncertainties are based only on the attitude determination uncertainties. Therefore, for the solar flux, the uncertainty is estimated at 4% (1σ) for both methods. For the eclipse phase and terrestrial flux, the TRIAD method allows us to compute the flux with around 19% uncertainty, although MEKF allows for around 13% accuracy (1σ). The MEKF method brings improvements. but it also presents limitations. The algorithm loads the data on-ground from in-orbit data to compute the CubeSat attitude. However, the method relies on the need to predict the state vector at the next iteration from the previous measurements.

**Figure 15:**
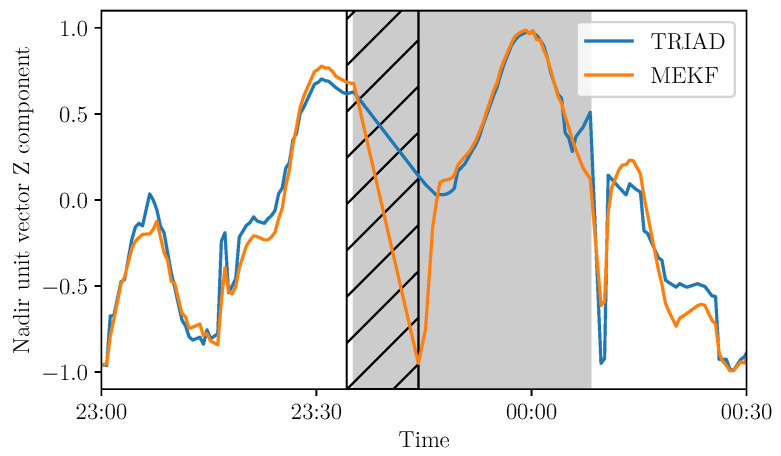
Nadir Z component in the satellite frame based on the TRIAD and MEKF methods—missing values are delimited with hatches for a 10 min duration on 3 February 2021.

Therefore, it is important that those two steps are not too far in time given the chosen sampling rate. Rarely, but not impossibly, the inputs data can be missing due to a single-event upset (SEU) or missing values in the communication process. An example of this appears in Figure 15.

After the missing values, the first estimations of the nadir component from the MEKF method seem to be very different from the TRIAD estimations. Data gaps cause the poor prediction of the next iteration. This issue can be solved by implementing the algorithm on-board the satellite to avoid missing values due to communication issues. To do so, the computations should be executed on the on-board computer. Computational resources can be limited in-orbit. Therefore, an optimized algorithm was developed to reduce the required computations and is described in [15].

This section has presented the results of the methods. The advantages and limitations can be summarized as follows. The use of infrared sensors instead of photodiodes in an eclipse allows us to greatly reduce the measurement uncertainty. This is still recent and rarely developed in the literature (Table 1, such as in [12] or [19]), but its use could be validated in orbit. Moreover, the use of the Kalman filter allowed us to increase the accuracy of the attitude restitution and is relatively computationally efficient compared to the Unscented Kalman Filter presented in [19]. However, certain limitations are present due to difficulties of a making predictions due to a lack of data.

## 5. Conclusions

UVSQ-SAT CubeSat aims to validate innovative technologies in-orbit for a future constellation to study the Earth’s energy imbalance. UVSQ-SAT has been in orbit since 24 January 2021. To improve the UVSQ-SAT’s reflected solar radiation and outgoing longwave radiation measurements at the top of the atmosphere, the UVSQ-SAT’s attitude must be accurately known. Two different methods were implemented to determine the UVSQ-SAT CubeSat’s attitude, and they are based on real data from space observations.

The first method developed is based on the TRIAD algorithm. The accuracy of the UVSQ-SAT attitude knowledge obtained with the TRIAD method is close to ±3${}^{{}^{\circ}}$ (at one σ) in sunlight. During eclipse periods, the accuracy of the UVSQ-SAT attitude knowledge is ±14${}^{{}^{\circ}}$ (at one σ). In this observation phase, accurate knowledge of the CubeSat attitude is more difficult to obtain. This mode of operation with other sensors (Earth radiative sensors instead of solar visible photodiodes) brings limits. Moreover, the TRIAD method does not correct all measurement noises.

The MEKF method allows us to estimate and correct instrument noise. It performs the real-time calibration of the UVSQ-SAT gyrometer. The MEKF method computes the UVSQ-SAT attitude knowledge with an accuracy similar to TRIAD in sunlight, but with an accuracy of ±10${}^{{}^{\circ}}$ during eclipse periods (at one σ). There are limitations to the MEKF method, such as the lack of continuous data. This is a limiting factor since it leads to large divergence errors. The prediction becomes better when the time between two measurements is short. Then, the linearization becomes more realistic. Reducing the time-step could be beneficial for future CubeSats, and ground-based tests in near-space conditions would be recommended to optimize the various parameters.

The methods presented on this manuscript are based on direct measurements (TRIAD) or Kalman filters (MEKF). Another approach would be to use neural networks to determine the UVSQ-SAT satellite’s attitude as described in [31]. The neural network will be implemented and trained in-orbit in sunlight to improve the attitude determination accuracy during eclipse periods. The training will be based on the previously described methods, and the performance of the new method will be evaluated to assess the ability of the method to be implemented for the future satellites of the constellation.

## Figures and Tables

**Table 1:** Studies related to attitude determination (simulation and in-orbit observations).

Reference	Method (Instruments)	Goal	Results/Remarks
[4]	Simulation	Attitude determination (AD) based solely on magnetometer	Converges from initial attitude errors of maximum 60${}^{{}^{\circ}}$ and with an attitude accuracy of 1${}^{{}^{\circ}}$ (1σ) or better
[5]	Observation (Rossi X-ray Timing Explorer satellite calibration maneuvers, Terra and Wide-Field Infrared Explorer mission, Upper Atmosphere Research Satellite (UARS))	On-orbit calibration of satellite gyroscopes	Methods comparison (attitude accuracy below 1${}^{{}^{\circ}}$). The Delta-bias algorithm gives slightly less accurate results than the Davenport and BICal algorithms
[6]	Simulation	Absolute alignment calibration of a system comprising two star trackers, an inertial sensor assembly (ISA) of three fiber-optic gyros, and an imaging instrument based on Alignment Kalman Filter (AKF)	AKF is effective to estimate absolute misalignments and gyro calibration parameters
[7]	Simulation	AD using an Extended Kalman Filter (EKF), which applies the albedo model with a magnetometer and sun sensor	Attitude accuracy below 1${}^{{}^{\circ}}$
[8]	Simulation and Observation (Total Ozone Mapping Spectrometer (TOMS))	Modeling the albedo for Sun/Earth sensor used in attitude determination	Albedo compensation in attitude estimation, improves the maximum error from 9.9${}^{{}^{\circ}}$ to 1.9${}^{{}^{\circ}}$
[9]	Simulation	AD using Unscented Kalman Filter (UKF) based only on magnetometer	The attitude estimation accuracies are below 0.5${}^{{}^{\circ}}$ after convergence
[10]	Simulation (PROBA-2 Spacecraft scenarios)	Navigation system for magnetic-only orbit and attitude estimation using the square-root Unscented filter (MAGSURF)	RSS attitude error of less than 1.4${}^{{}^{\circ}}$ and a time of convergence of less than 2 orbits
[11]	Simulation	Attitude and rate estimation algorithm using EKF based only on geomagnetic field data	Filter converges within the +/−8${}^{{}^{\circ}}$ range for any initial attitude error
[12]	Simulation (Radio Aurora Explorer satellites (3U CubeSat))	AD based on gyros, magnetometers, coarse sun sensors, and an EKF	In the sun, the angular uncertainty is between 2${}^{{}^{\circ}}$ and 3${}^{{}^{\circ}}$, and in eclipse, the uncertainty increases to between 7${}^{{}^{\circ}}$ and 8${}^{{}^{\circ}}$
[13]	Simulation	AD using two-step EKF based on a magnetometer only	Attitude accuracies of less than 1${}^{{}^{\circ}}$
[3]	Observation (Radio Aurora Explorer satellites (3U CubeSat))	Photodiodes calibration and AD from EKF/UKF with albedo model based on the calibrated photodiodes, three-axis magnetometer and gyrometer	Angular improvement of 10${}^{{}^{\circ}}$ in sun vector from the photodiodes, and below 1${}^{{}^{\circ}}$ accuracy on the attitude determination
[14]	Simulation	AD via a robust Adaptive Kalman Filter based on magnetometer and gyro measurement	Precision of traditional EKF is about 0.2${}^{{}^{\circ}}$, and the maximum estimate error of the robust adaptive filter is 0.1${}^{{}^{\circ}}$
[15]	Simulation and Observation (experimental data with on-ground nano-satellite)	Gain-scheduled EKF (GSEKF) to reduce the computational requirement in the nanosatellite attitude determination process	Attitude accuracy below 0.2${}^{{}^{\circ}}$ during the entire orbital period. Computation time could be reduced by 86.29% and 89.45%
[16]	Simulation	Magnetometer calibration with Hyper least square (HyperLS) estimator for ellipsoid fitting, then utilized for attitude determination via non-linear colored noise filters of EKF, simplex UKF and cubature Kalman filter	Attitude accuracy below 1${}^{{}^{\circ}}$ for simplex UKF
[17]	Observation (images taken from International Space Station (ISS))	AD utilizing color earth images taken with visible light camera	Attitude accuracy is about few degrees or less
[18]	Simulation	Heat attitude model for satellite attitude determination	Attitude accuracy between 0.2 ${}^{{}^{\circ}}$ to 5${}^{{}^{\circ}}$
[19]	Simulation	AD method based on an UKF, using a gyrometer, a magnetometer and solar panels as a sun sensor	The UKF has shown precision in Euler angles of about 1.1${}^{{}^{\circ}}$, which is better than for EKF. UKF has a considerably longer processing time compared to EKF
[20]	Simulation and Observation (experimentation on-ground set up)	Thermal imaging sensors to determine attitude of the Sun and the horizon by employing a homogeneous array of such detectors	Angular accuracy below 1${}^{{}^{\circ}}$

**Table 2:** Definition of the true positive, false negative, false positive, and true negative.

Definition		Attitude Determination	
		Facing the Sun	Not facing the Sun
**UVS**	Facing the Sun	*True Positive (TP)*	*False Negative (FN)*
	Not Facing the Sun	*False Positive (FP)*	*True Negative (TN)*

## Data Availability

Not applicable.

## References

[1] Meftah M., Damé L., Keckhut P., Bekki S., Sarkissian A., Hauchecorne A., Bertran E., Carta J.P., Rogers D., Abbaki S. (2020). UVSQ-SAT, a pathfinder cubesat mission for observing essential climate variables. Remote Sens..

[2] Meftah M., Boutéraon T., Dufour C., Hauchecorne A., Keckhut P., Finance A., Bekki S., Abbaki S., Bertran E., Damé L. (2021). The UVSQ-SAT/INSPIRESat-5 CubeSat Mission: First In-Orbit Measurements of the Earth’s Outgoing Radiation. Remote Sens..

[3] Springmann J.C., Cutler J.W. (2014). On-orbit Calibration of Photodiodes for Attitude Determination. J. Guid. Control. Dyn..

[4] Psiaki M.L., Martel F., Pal P.K. (1990). Three-axis attitude determination via Kalman filtering of magnetometer data. J. Guid. Control. Dyn..

[5] Hashmall J.A., Radomski M., Sedlak J. On-orbit calibration of satellite gyroscopes. Proceedings of the Astrodynamics Specialist Conference.

[6] Pittelkau M.E. (2001). Kalman Filtering for Spacecraft System Alignment Calibration. J. Guid. Control. Dyn..

[7] Theil S., Appel P., Schleicher A. (2003). Low Cost, Good Accuracy—Attitude Determination Using Magnetometer and Simple Sun Sensor. https://digitalcommons.usu.edu/smallsat/2003/All2003/81/.

[8] Bhanderi D., Bak T. Modeling Earth Albedo for Satellites in Earth Orbit. Proceedings of the AIAA Guidance, Navigation, and Control Conference and Exhibit.

[9] Ma G.-F., Jiang X.-Y. Unscented Kalman Filter for Spacecraft Attitude Estimation and Calibration Using Magnetometer Measurements. Proceedings of the 2005 International Conference on Machine Learning and Cybernetics.

[10] Côté J., De Lafontaine J. (2008). Magnetic-only orbit and attitude estimation using the Square-Root Unscented Kalman Filter: Application to the PROBA-2 spacecraft. AIAA Guidance, Navigation and Control Conference and Exhibit.

[11] Filipski M.N., Varatharajoo R. (2010). Evaluation of a spacecraft attitude and rate estimation algorithm. Aircr. Eng. Aerosp. Technol..

[12] Springmann J.C., Sloboda A.J., Klesh A.T., Bennett M.W., Cutler J.W. (2012). The attitude determination system of the RAX satellite. Acta Astronaut..

[13] Searcy J.D., Pernicka H.J. (2012). Magnetometer-Only Attitude Determination Using Novel Two-Step Kalman Filter Approach. J. Guid. Control. Dyn..

[14] Zeng Z., Zhang S., Xing Y., Cao X. (2014). Robust Adaptive Filter for Small Satellite Attitude Estimation Based on Magnetometer and Gyro. Abstr. Appl. Anal..

[15] Pham M.D., Low K.S., Goh S.T., Chen S. (2015). Gain-scheduled extended Kalman filter for nanosatellite attitude determination system. IEEE Trans. Aerosp. Electron. Syst..

[16] Kiani M., Pourtakdoust S.H., Sheikhy A.A. (2015). Consistent calibration of magnetometers for nonlinear attitude determination. Meas. J. Int. Meas. Confed..

[17] Koizumi S., Kikuya Y., Sasaki K., Masuda Y., Iwasaki Y., Watanabe K. (2018). Development of Attitude Sensor using Deep Learning. AIAA/USU Conference on Small Satellites.

[18] Labibian A., Pourtakdoust S.H., Alikhani A., Fourati H. (2018). Development of a radiation based heat model for satellite attitude determination. Aerosp. Sci. Technol..

[19] Baroni L. (2020). Attitude determination by unscented Kalman filter and solar panels as sun sensor. Eur. Phys. J. Spec. Top..

[20] Kapás K., Bozóki T., Dálya G., Takátsy J., Mészáros L., Pál A. (2021). Attitude Determination for Nano-Satellites—I. Spherical Projections for Large Field of View Infrasensors. Exp. Astron..

[21] Mimasu Y., Narumi T., Van der Ha J. (2008). Attitude Determination by Magnetometer and Gyros During Eclipse.

[22] Alken P., Thébault E., Beggan C.D., Amit H., Aubert J., Baerenzung J., Bondar T.N., Brown W.J., Califf S., Chambodut A. (2021). International Geomagnetic Reference Field: The thirteenth generation. Earth Planets Space.

[23] Black H.D. (1964). A passive system for determining the attitude of a satellite. AIAA J..

[24] Markley F.L., Mortari D. (2000). How to estimate attitude from vector observations. Adv. Astronaut. Sci..

[25] Bar-Itzhack I.Y., Harman R.R. (1997). Optimized TRIAD Algorithm for Attitude Determination. J. Guid. Control. Dyn..

[26] Bar-itzhack I.Y., Meyer J. (1976). On the Convergence of Iterative Orthogonalization Processes. IEEE Trans. Aerosp. Electron. Syst..

[27] Kalman R.E. (1960). A New Approach to Linear Filtering and Prediction Problems. Trans. ASME-Basic Eng..

[28] Kalman R.E., Bucy R.S. (1961). New Results in Linear Filtering and Prediction Theory. J. Basic Eng..

[29] Markley F.L., Crassidis J.L. (2014). Static Attitude Determination Methods. Fundamentals of Spacecraft Attitude Determination and Control.

[30] Lefferts E.J., Markley F.L., Shuster M.D. (1982). Kalman Filtering for Spacecraft Attitude Estimation. J. Guid. Control. Dyn..

[31] Finance A., Meftah M., Dufour C., Boutéraon T., Bekki S., Hauchecorne A., Keckhut P., Sarkissian A., Damé L., Mangin A. (2021). A New Method Based on a Multilayer Perceptron Network to Determine In-Orbit Satellite Attitude for Spacecrafts without active ADCS like UVSQ-SAT. Remote Sens..

